# Advanced Therapeutics, Vaccinations, and Precision Medicine in the Treatment and Management of Chronic Hepatitis B Viral Infections; Where Are We and Where Are We Going?

**DOI:** 10.3390/v12090998

**Published:** 2020-09-07

**Authors:** Ganesh Selvaraj Duraisamy, Dattatry Bhosale, Ivana Lipenská, Ivana Huvarova, Daniel Růžek, Marc P. Windisch, Andrew D. Miller

**Affiliations:** 1 Veterinary Research Institute, Hudcova 70, CZ-62100 Brno, Czech Republic; duraisamy@vri.cz (G.S.D.); bhosale@vri.cz (D.B.); lipenska.ivana@gmail.com (I.L.); huvarova@vri.cz (I.H.); ruzekd@paru.cas.cz (D.R.); 2 Institute of Parasitology, Biology Centre of the Czech Academy of Sciences, Branisovska 31, CZ-37005 České Budějovice, Czech Republic; 3 Applied Molecular Virology Laboratory, Institut Pasteur Korea, 696 Sampyeong-dong, Bundang-gu, Seongnam-si, Gyeonggi-do 463-400, Korea; marc.windisch@ip-korea.org; 4 Division of Bio-Medical Science and Technology, University of Science and Technology, Daejeon 305-350, Korea; 5 Department of Chemistry and Biochemistry, Mendel University in Brno, Zemědělská 1, Černá Pole, CZ-61300 Brno, Czech Republic; 6 KP Therapeutics (Europe) s.r.o., Purkyňova 649/127, CZ-61200 Brno, Czech Republic

**Keywords:** lipid-based nanoparticles, biophysics, nanomedicine, nanotechnology, plasmid DNA, RNA interference, small interfering RNA, active pharmaceutical ingredient, precision medicine, personalized medicine, precision therapeutics approach, hepatitis B virus

## Abstract

The management of chronic hepatitis B virus (CHB) infection is an area of massive unmet clinical need worldwide. In spite of the development of powerful nucleoside/nucleotide analogue (NUC) drugs, and the widespread use of immune stimulators such as interferon-alpha (IFNα) or PEGylated interferon-alpha (PEG-IFNα), substantial improvements in CHB standards of care are still required. We believe that the future for CHB treatment now rests with advanced therapeutics, vaccination, and precision medicine, if all are to bring under control this most resilient of virus infections. In spite of a plethora of active drug treatments, anti-viral vaccinations and diagnostic techniques, the management of CHB infection remains unresolved. The reason for this is the very complexity of the virus replication cycle itself, giving rise to multiple potential targets for therapeutic intervention some of which remain very intractable indeed. Our review is focused on discussing the potential impact that advanced therapeutics, vaccinations and precision medicine could have on the future management of CHB infection. We demonstrate that advanced therapeutic approaches for the treatment of CHB, in the form of gene and immune therapies, together with modern vaccination strategies, are now emerging rapidly to tackle the limitations of current therapeutic approaches to CHB treatment in clinic. In addition, precision medicine approaches are now gathering pace too, starting with personalized medicine. On the basis of this, we argue that the time has now come to accelerate the design and creation of precision therapeutic approaches (PTAs) for CHB treatment that are based on advanced diagnostic tools and nanomedicine, and which could maximize CHB disease detection, treatment, and monitoring in ways that could genuinely eliminate CHB infection altogether.

## 1. Unmet Need in Treatment

Hepatitis B virus (HBV) infections are endemic and a major human health problem in many countries worldwide [1]. Chronic hepatitis B virus (CHB) infection affects more than 350 million people and more than 780,000 persons die annually due to virus-related secondary diseases such as liver cirrhosis (LC) and the development of hepatocellular carcinoma (HCC) [2]. HBV is a blood-borne viral disease transmitted person to person by transfusion with contaminated blood, body fluids from sexual contact, body piercing, unsafe needle injection practices, and infected mother to newborn child transfers [2]. Unfortunately, newborn babies are at a very high risk of contracting HBV (90%) from their infected mothers peri- and postpartum and then become chronic carriers in their own right. There is also horizontal transmission amongst young children by unknown mechanisms, which is higher for children under five years (25–30%) than for adolescents/adults (<5%) [3]. Accordingly, there are now extensive efforts aimed at improving the diagnosis of HBV infections and of HBV-associated diseases [4]. In addition, there are extensive efforts aimed at the realization of better treatments of CHB infections. Unfortunately, the complexity of the underlying mechanisms that promote HBV persistence in vivo have ensured that the creation and development of effective treatments against CHB infections remains a great challenge [4]. Therefore, the treatment of CHB infections is still today an area of high-unmet medical need.

### 1.1. Virus Replication Cycle

**Figure 1:**
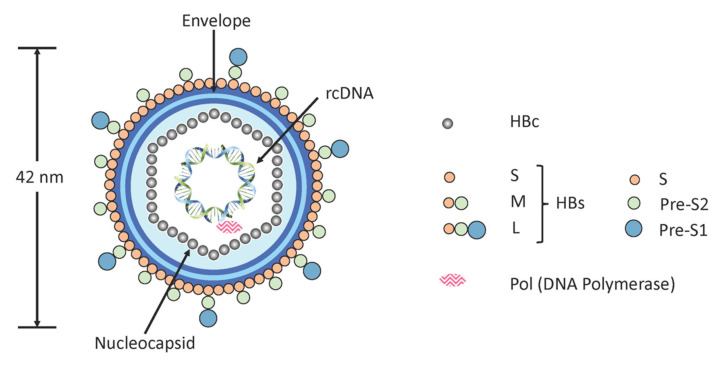
Schematic diagram of a HBV particle. The illustrated HBV infectious virion has the indicated main features as labelled with dimensions as shown.

The problem of treating HBV starts with the nature of the virus itself. HBV is a hepatotropic, non-cytopathic family member of the *hepadnaviridae* comprising a 3.2kb partly double-stranded, relaxed circular DNA (rcDNA) genome and viral DNA polymerase (pol) condensed into a nucleocapsid by hepatitis B core (HBc) proteins. The rcDNA of HBV comprises a DNA negative (anti-sense) strand encompassing the entire genome, with a single, fixed-site ‘nick’, which is partially hybridised to an incomplete DNA positive (sense) strand. This nucleocapsid is encompassed by an outer lipid/protein envelope consisting mainly of three envelope proteins, otherwise identified as large, medium and small hepatitis B surface (L-HBs, M-HBs, and S-HBs) proteins that are known collectively as hepatitis B surface (HBs) proteins [5] (Figure 1).

**Figure 2:**
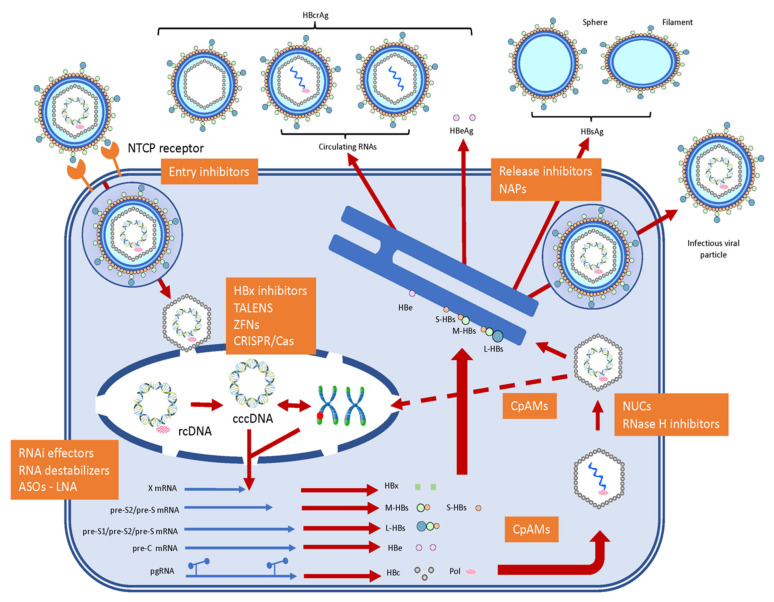
Schematic diagram of HBV infection pathway. The pathway of infection involves NTCP receptor-mediated uptake of HBV particles into hepatocytes, followed by controlled endo-osmolysis that enables the nuclear capsid to enter the cell nucleus where rcDNA can be processed into fully functional cccDNA. This is either propagated into pgRNA for new virus particle production or integrated into host cell chromosomes. The pgRNA has multiple functions to initiate the re-synthesis of viral DNA, plus originate pre-C mRNA and all other sub viral mRNAs that code for all the main viral proteins. In the case of the first function, pgRNA is encapsulated with viral DNA polymerase (Pol) using HBc proteins to form immature nucleocapsids within which pgRNA is subject to reverse transcription by Pol to form complementary single-stranded DNA [negative (anti-sense)]. This is then nicked during reverse transcription as positive (sense) strand DNA is being partially generated and rcDNA reformed. Packaged rcDNA in mature nucleocapsid is then processed through the endoplasmic reticulum (ER) of a host hepatocyte leading to exocytosis of mature infectious HBV viral particles. The different classes of active pharmaceutical agents (APIs) that can modulate the infection cycle are as follows: entry inhibitors that act on NTCP mediated entry; anti-cccDNA agents such as HBx inhibitors, transcription activator-like effector nucleases (TALENs), zinc finger nucleases (ZFNs), and clustered regularly interspaced short palindromic repeat (CRISPR)/CRISPR-associated endonuclease (Cas); anti-mRNA agents such as RNA interference (RNAi) effectors, RNA destabilizers, and antisense oligonucleotides (ASOs)–locked nucleic acids (LNAs); anti-nucleocapsid assembly agents such as core protein allosteric modulators (CpAMs); reverse transcriptase inhibitors such as nucleoside/nucleotide analogues (NUCs) or RNaseH inhibitors; release inhibitors including nucleic acid polymers (NAPs).

The viral replication cycle begins with cell entry that involves hepatocyte receptor-specific targeting of the sodium taurocholate co-transporting polypeptide (NTCP) receptor [6]. Virus particle-NTCP receptor interactions enable virions to enter hepatocytes by receptor-mediated endocytosis during which nucleocapsids are released into the cytoplasm. These nucleocapsids then interact with importin-α or -β for transport into hepatocyte cell nuclei via nuclear pore complexes, thus commencing the infection process (Figure 2). In the first stage, individual rcDNAs are repaired into covalently closed circular DNAs (cccDNAs), which are then packaged into chromatin by histone and non-histone proteins [7,8]. Fundamentally, such packaged cccDNAs can be thought of as viral minichromosomes that are responsible for viral persistence in the nuclei of infected cells. These cccDNAs are the only known template for the transcription of pre-genomic RNA (pgRNA), from which viral DNA is eventually resynthesized by reverse transcription [7]. These cccDNAs also serve for the transcription of pre-C mRNA, and all other sub-genomic mRNAs that code for the main viral proteins [8]. The main proteins concerned are L-HBs, M-HBs, S-HBs, HBc, Pol, the hepatitis B x (HBx) protein, and the hepatitis B e (HBe) protein. L-HBs proteins are translated from the longer pre-S1/pre-S2/pre-S mRNA transcript, M-HBs, and S-HBs are translated both from a shorter pre-S2/pre-S mRNA transcript. HBe proteins are translated from the pre-C mRNA transcript, while HBc and Pol proteins are preferentially translated from pgRNA [9,10]. Finally, the HBx protein is translated from the X open reading frame mRNA transcript.

Functionally speaking, HBe protein is translated from a longer reading frame than HBc protein, then undergoes extensive post-translational modification, and is finally secreted into the bloodstream as HBe antigen (HBeAg), one of the main immunoactive biomarkers for HBV infection [10]. By contrast, the HBx protein remains intracellular, where it targets a complex involving the structural maintenance of chromosomes protein 5 (SMC5) and protein 6 (SMC6) on cccDNA. Binding of HBx to the SMC5/6 complex, tags this complex for proteolysis by the proteasome and lifts SMC5/6-mediated inhibition of cccDNA transcription [10]. Otherwise, HBc proteins initially form a nucleation complex with pgRNA-bound Pol protein, in order to trigger complete nucleocapsid assembly. Thereafter, Pol performs reverse transcription of pgRNA yielding an encapsidated complementary DNA negative (anti-sense) strand, which is then used as a template for DNA positive (sense) strand synthesis and rcDNA reformation [9]. Following this, newly mature nucleocapsids either recycle back to hepatocyte cell nuclei, to maintain an active pool of cccDNA, or else bud into the lumen of the cell endoplasmic reticulum (ER) where they acquire lipid/protein envelopes largely comprised of L-HBs, M-HBs, and S-HBs proteins. The resulting immature viral particles then undergo a further maturation process, that involves surface protein glycan modifications, as they pass through the Golgi complex to the trans-Golgi. Mature, infectious viral particles are then exported/released from host hepatocyte cells by exocytosis (Figure 2) [7,10].

This replication cycle is accompanied by the export/release of a range of incomplete sub-viral particles (SVPs) alongside infectious viral particles [10]. These, in combination with the tolerogenic liver environment, help create a highly suppressed immunological environment that ensures CHB infections are both durable and very difficult to eradicate. The most abundant of these exported SVPs are particulate forms of viral envelopes (sphere and filament), formed with HBs proteins, that are known collectively in the bloodstream as HBs antigen (HBsAg), a main immunoactive biomarker for HBV infection in conjunction with HBeAg. Other exported SVPs that only encapsidate mRNAs, not rcDNA, or lack nucleocapsid altogether, are known collectively as hepatitis B core-related antigen (HBcrAg). SVPs that only encapsidate mRNAs, alongside mRNA containing exosomes, are known as “circulating RNAs” (Figure 2). Importantly, HBcrAg can be detected in serum, even when HBV DNA cannot [10].

### 1.2. Functional Cure

Anti-HBV treatments are administered to patients presenting a detectable viral load in the blood and liver inflammation, characterized by fluctuating or stably elevated levels of viral replication and of alanine transferase (ALT). Such CHB patients are also defined as being either HBeAg-positive or -negative. A functional cure should comprise a comprehensive amelioration of these disease features and include remission of liver inflammation, plus a clear decrease in the risks of LC and HCC. Therefore, treatment guidelines have focused on the mediation of substantial and sustained reductions in HBsAg blood pool levels, with or without the development of anti-HBs antibodies [10]. Accordingly, the goal of any new treatment/therapeutic approach must be to maximize the rate of functional cure. However, the HBV replication cycle is so complex that it is difficult to imagine how any one active pharmaceutical ingredient (API) might be designed and created to tackle HBV infection on its own, as past experience is already showing (see Section 2 of this review).

### 1.3. Genotypes of HBV

Functional cure of CHB infections should be further complicated by the existence of HBV genotypes categorized by genetic divergence in the full-length HBV genome sequence involving a genetic variation of 4–8% in the S gene level [11]. There are now known to be a least 10 such genotypes (A, B, C, D, E, F, G, H, I, and J) according to gene sequence variations (Table 1).

Genotype A is the most found prevalent in Europe, North America, Sub-Saharan Africa, and Western Africa, whereas genotypes B and C are more predominant in Asia [12]. In China, the overall presence of HBsAg in people is >7%, and more than 60% of LC and 80% of HCC cases are caused by CHB. Therefore, the disease burden is particularly severe in China. The D genotype is mainly found in the Mediterranean area, the Middle East, and India, while the E genotype is most found in Sub-Saharan Africa [13,14]. On the other hand, the F and H genotypes appear to be most common in Central and South America [15], whereas the G genotype is often found in co-infections with other genotypes, and can be found in France, Germany, United States, and Mexico. Finally, I and J genotypes have only just been determined according to genomic divergence and have yet to be more fully evaluated [12]. The I genotype was identified in Vietnam and Laos involving in an inter-genotypic combination among A, C, and G [16]. The J genotype was identified from Japanese patients [17] and has a direct relationship with the gibbon/orangutan and human C genotype. All these genetic variations have mainly arisen because of “errors” during virus replication. 

## 2. Currently Approved Treatments for CHB Infections

**Figure 3:**
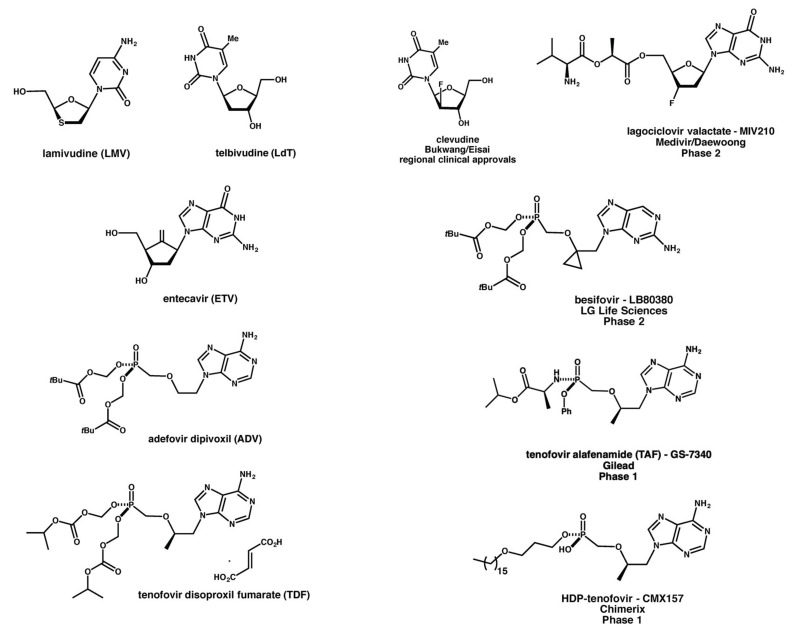
Chemical structures of major clinically approved NUCs. The figure is divided to show NUCs on market (left-hand column), and NUCs in development (right-hand column). Another NUC of note in development is AGX-1009, which is described as a TDF pro-drug.

Currently, two different therapeutic approaches have been approved by the Food and Drug Administration (FDA) to treat CHB patients. These comprise the use of:

1. interferon-alpha (IFNα) or PEGylated interferon-alpha (PEG-IFNα) and/or
2. direct-acting antivirals (DAAs) such as nucleoside/nucleotide analogues (NUCs) that include nucleoside analogues lamivudine (LMV), telbivudine (LdT) and entecavir (ETV), or nucleotide analogues adefovir dipivoxil (ADV), and tenofovir disoproxil fumarate (TDF) [10] (Figure 3).

### 2.1. Interferons

Conventional IFNα is an inducer of antiviral effects through the suppression of viral DNA synthesis and by stimulation of antiviral enzyme production. Treatment with IFNα results in the clearance of virus-infected cells, enabling a proportion of CHB patients to achieve a sustained virologic response (SVR) post-treatment. Many studies describe how IFNα treated CHB patients exhibit an SVR of up to 37%, with a mean loss rate of 33% in HBeAg and of 8% in HBsAg levels [18]. SVRs in IFNα treated CHB patients, whether child or adult, are the same when high ALT serum levels exist during treatment, but post-treatment SVRs reduce to 10% amongst those CHB patients with normal serum ALT levels [19]. Other factors that impact on SVRs following IFNα treatment include patient age, low serum levels of HBV DNA, early infection (even from birth), patient naïvety to IFNα treatment, the presence of pre-core HBV mutations, chronicity, plus sex and co-infection with human immunodeficiency virus (HIV). HBV genotype and patient geographical distributions will also have an impact (Table 2). In a cohort of HBeAg-positive LC patients, long-term follow-up data after IFNα treatment showed that the rate of HBeAg suppression, compared with a control group, was similar (67% vs. 60%, respectively) although the ALT normalization rates (62% vs. 47%) and HBsAg loss rates (23% vs. 3%) were better [19].

Treatment with PEG-IFNα was introduced to prolong the effective half-life of IFNα, reduce functional dose levels, increase efficacy compared with IFNα alone, and lower side effects [20]. The hope was that PEG-IFNα would deliver on a more sustained, stable antiviral effect, and hence improve SVRs. PEG-IFNα is known to act more specifically as an immune modulator to increase the cellular immune responses against hepatocyte cells infected with HBV [20]. There are two categories of PEG-IFNα known as PEG-IFNα-2a and PEG-IFNα-2b, according to the mono-PEGylated IFNα isomers involved. Treatment with PEG-IFNα-2b in HBeAg-positive CHB patients was found to result in a loss of 27% in HBeAg levels in patients plus a 25% loss in serum HBV DNA levels after 48 weeks of treatment [21]. In HBeAg-negative CHB patients, those treated with PEG-IFNα were found to have HBsAg serum levels suppressed even up to three years post-treatment, indicative of a potent SVR. Nevertheless, randomized studies suggest that PEG-IFNα effects are typically best in HBeAg-positive rather than in HBeAg-negative CHB patients [22]. For example, long-term treatment with PEG-IFNα in HBeAg-positive patients with CHB led to viral suppression in 10–40% of patients, with an HBeAg loss of approximately 30–35%, accompanied by a normalization of ALT levels in 35–50% patients. An HBsAg loss was then observed in approximately 5% of patients 6 months after treatment cessation and in 10% of patients 3 years post-treatment. As with IFNα treatment, the benefits of PEG-IFNα treatment will vary with HBV genotype and patient geographical distributions (Table 2). Overall, PEG-IFNα is not effective in all CHB patients by any means and maybe much less tolerated than IFNα. Therefore, the treatment applications of PEG-IFNα are necessarily much more limited than would be desirable. In general, treatments involving IFNα and PEG-IFNα are associated with significant side effects, suboptimal response rates in patients with advanced liver diseases, and can be associated with fatal decompensation in patients with LC [22].

### 2.2. Nucleoside/Nucleotide Analogues

NUCs are essentially small molecule drugs (<500 Da in molecular weight) that directly inhibit the reverse transcriptase activity of the HBV DNA polymerase and hence reduce virion production [10,23,24]. For example, after 3 years of continuous treatment with LMV (100 mg daily), 23 out of 58 patients achieved a rate of HBeAg suppression of approximately 40%, while in patients with baseline serum ALT >2× upper limit of normal, the rate of HBeAg suppression was 65% (17 out of 26) [23,24]. Unfortunately, long-term LMV treatment leads to the development of drug resistance owing to induced HBV DNA polymerase mutations. Resistance rates might be up to 20% after a year of treatment, increasing up to 70% after five years of treatment [23,24]. Similarly, resistance rates can be up to 22% in HBeAg-positive, and 9% in HBeAg-negative CHB patients treated with LdT [23,24]. Accordingly, LMV and LdT treatment are nowadays not preferred due in part to their weak antiviral potencies and the high frequencies with which drug resistance develops. In addition, cases of mitochondrial toxicity have emerged, with LMV or LdT treatment, that are generally observed as myopathies, neuropathies, or lactic acidoses [24], although the incidence of LMV-induced lactic acidosis during treatment is rare [25].

On the other hand, long-term therapy with second generation NUC, ETV, has been shown to result in durable and increasing viral suppression, with undetectable levels of HBV DNA (<300 copies/mL) achieved in 94% of HBeAg-positive patients over 5 years of treatment, and in 95% of HBeAg-negative patients over three years of treatment [26]. Otherwise, TDF has demonstrated superior antiviral efficacy over ADV in HBeAg-positive and HBeAg-negative CHB patients. Durable and increasing viral suppression was observed over four years of treatment with HBV DNA found undetectable (<400 copies/mL) in 96% of HBeAg-positive patients and in 99% of HBeAg-negative patients. Moreover, in HBeAg-positive patients, HBeAg losses of up to 29% occurred in 41% of patients, while cumulative HBsAg losses were 11%. TDF was well tolerated over this treatment period [23]. However, HBeAg suppression rates post five years of TDF, or ETV treatment (40% and 44%, respectively) are far from perfect. Unfortunately, long-term treatment with second generation NUCs can also lead to resistance rates of up to 30% [27]. So too, there is a high risk of cross-resistance developing that is now causing major problems for the ongoing treatment of CHB patients [27]. In addition, nephrotoxicity has been observed in patients treated with TDF [28], while risk factors include old age, low body weight (<60 kg), male gender, pre-existing renal impairment, concomitant use of nephrotoxic medications, HCV coinfection, gene polymorphisms of transporter proteins, and high levels of plasma TDF (>160 ng/ml) [29,30,31]. TDF treatment can also reduce bone mineral density (BMD) in some patients [32]. Recently, tenofovir alafenamide (TAF) is proving to be a promising alternative to TDF. TAF treatment appears to cause less adverse effects and is applicable in the treatment of CHB patients who are at risk of renal and/or bone complications [33]. TAF has also been shown to be a more potent inhibitor of HBV replication than TDF at low doses and is more effective at returning ALT levels to within normal limits [34]. Furthermore, TAF treatment is less prone to reducing BMD in either HBeAg-positive or HBeAg-negative patients [35]. In general, treatment and the development of resistance during long-term treatment with NUCs will also be influenced by HBV genotype and patient geographical distributions. Once again, the known modifying impact of HBV genotypes on NUC treatment outcomes are summarized (Table 2). Furthermore, there are clear indications that even when treated with the second generation NUCs, such as ETV and TDF, patients with highly impaired liver functions can develop lactic acidosis [25,36]. Currently, the clinical relevance of TAF treatment is yet to be fully established and further studies are required to follow up the long-term use of this prodrug [37].

Disappointment with monotherapies has led to the most recent adoption of combination approaches in clinic involving both PEG-IFNα and NUCs to increase the efficacy of treatment, avoid or suppress drug-resistance development, reduce toxicity, shorten the duration of treatment, improve antigen suppression and promote the restoration of immune responses against HBV. The use of combinations of PEG-IFNα with LMV or LdT have reportedly resulted in increased antigen suppression, although absolute rates of suppression were not as great as might have been hoped for, suggesting that these combinations are not optimal for effective treatment [27]. Alternative combinations of PEG-IFNα with ETV resulted in more impressive antigen suppression with losses in HBeAg and HBsAg of 68.2% and 40.9%, respectively [38]. Moreover, it has been suggested that combinations of either ETV or TDF followed by PEG-IFNα or vice versa could give more sustained suppressions of HBeAg and HBsAg levels both during and after treatment. Other data suggest that the potency of combination therapy is less optimal in the case of HBV C and D genotype infections [38].

### 2.3. The Problem with Currently Approved Treatments

Current CHB treatments involve a number of different APIs, including PEG-IFNα, NUCs, and potentially other nucleoside/nucleotide reverse transcriptase inhibitors (NRTIs) [39]. All these approaches are intended to focus on the suppression of HBV replication to lower viremia and hence inhibit the development of fatal downstream liver diseases. Moreover, several studies have reported that NUCs and PEG-IFNα may inhibit HBV replication in patients, but the actual elimination of virus remains rare [40]. Such incomplete clinical coverage appears to be the result of API inadequacies during antiviral treatments, particularly with reference to cccDNA, plus treatment interruptions. Both make possible a rejuvenation of viral replication and the appearance of hepatic flares due to the exacerbation of CHB infection [40]. In addition, NUCs and/or PEG-IFNα have little or no direct impact on viral transcription or cccDNA and so there is a very high risk of reactivation of viral replication post-treatment, or the emergence of down-stream disease symptoms. For example, long-term treatment with NUCs can lead to reversion of LC, improved liver function, and prevent the need for liver transplantation. However, the risk of developing HCC is not removed [24], although the risk of contracting HCC may be lowered [41]. For similar reasons, the use of all these APIs typically fails to effect the functional cure of HBV infections with any degree of efficiency [40]. Therapeutic vaccines have been developed as an alternative to promote seroclearance of HBsAg, thereby reducing liver failure and increasing patient survival rates. Such approaches rely on focusing the potential of innate and specific immune reactions for substantial and directed effects against the HBV replication cycle. Unfortunately, this is difficult to achieve, therefore, reductions in viral infection levels remain modest, as measured by a 6–10% loss of HBsAg in patient sera after long-term treatments [24].

### 2.4. New Drugs in Pipeline

Since current CHB treatments offer little more than a means to prolong disease state management by sustained but only partial interventions in the HBV replication process [40]. Accordingly, pharmaceutical companies, small and large, have been very active in searching for novel APIs and therapeutic interventions that can selectively address all of the major aspects of the HBV replication cycle (Figure 2). How well these all work remains to be seen. What is clear, however, is that effective treatment of CHB probably requires that two clear challenges should be addressed in full. The first is the design and creation of APIs that can truly impact on the cccDNA replication intermediate, which is very stable and apparently quite refractory to external interventions. The other key challenge must be to overcome the immunosuppression of adaptive immune responses to HBV infection owing to the anergizing effect of HBeAg and HBsAg in the bloodstream on HBV-specific B- and T-cells during CHB infections. The large quantity of these viral antigens progressively causes alteration of functionality and deletion within these B- and T-cell populations [10]. Currently, the vast majority of novel APIs in pipeline are small molecules, including several new NUCs in development, as illustrated (Figure 3). Such small molecule anti-HBV APIs have recently been well-reviewed elsewhere [10,22,42], therefore these will not feature in the remainder of this review going forward. Instead, we shall now focus on the emergence of advanced therapeutics, vaccinations, and precision medicine approaches for the management and treatment of CHB infections.

## 3. Advanced Therapeutic Approaches for CHB Treatment

The standard of care for HBV treatment is clear, such that new and innovative advanced therapeutic approaches are essential to address the whole HBV replication cycle, and thereby achieve a functional cure. Therefore, of particular interest here is the ongoing design, creation and development of advanced therapeutic approaches for CHB treatment that either specifically target cccDNA and viral mRNAs to modulate the HBV replication cycle, and/or can overcome the anergizing effect of HBeAg and HBsAg on HBV-specific B and T-cells during CHB infections. Such advanced therapeutic approaches are intended not only to be highly effective following application but should avoid any requirement for indefinite administration [43]. The methods under review for treating CHB by targeting cccDNA and viral mRNAs might all be described as variants of gene therapy approaches, while those for overcoming immunosuppression might be described as targeted immune therapies [43]. Targeted immune therapies may also be complemented by therapeutic vaccination strategies.

### 3.1. Gene Therapy Approaches

**Figure 4:**
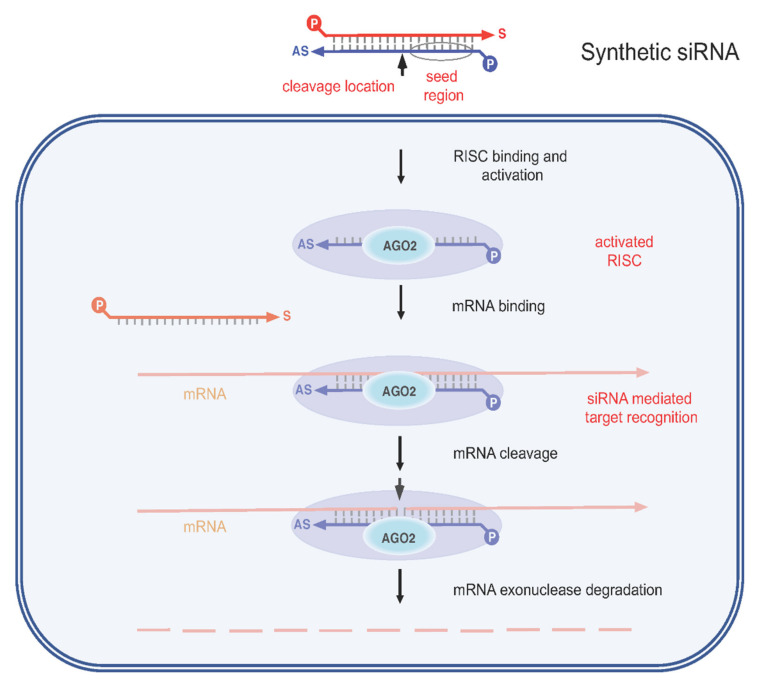
Schematic representation of a siRNA molecule and its interfering function. A given siRNA (top) has a well-defined structure: a short RNA duplex (19–23 bps) with two optional 2 nt overhangs on either 3′-end (frequently DNA), each strand is typically terminated with a 5′-phosphate and a 3′-hydroxyl group. Once introduced inside a cell cytoplasm, an siRNA is strand separated, and the guide strand (antisense, AS, strand in blue) is sequestered by an RNA-induced silencing complex (RISC) in a process involving the RISC Argonaute (Ago) protein (Ago2 in human cells), leading to RISC activation. The siRNA passenger strand (sense, S, strand in red) is discarded and is subsequently degraded. Otherwise, the Ago2 bound siRNA guide strand now enables activated RISC to bind to complementary target mRNA, a process that triggers the selective endo-nucleolytic cleavage of target mRNA, and the effective post-transcriptional knockdown of gene expression.

**Figure 5:**
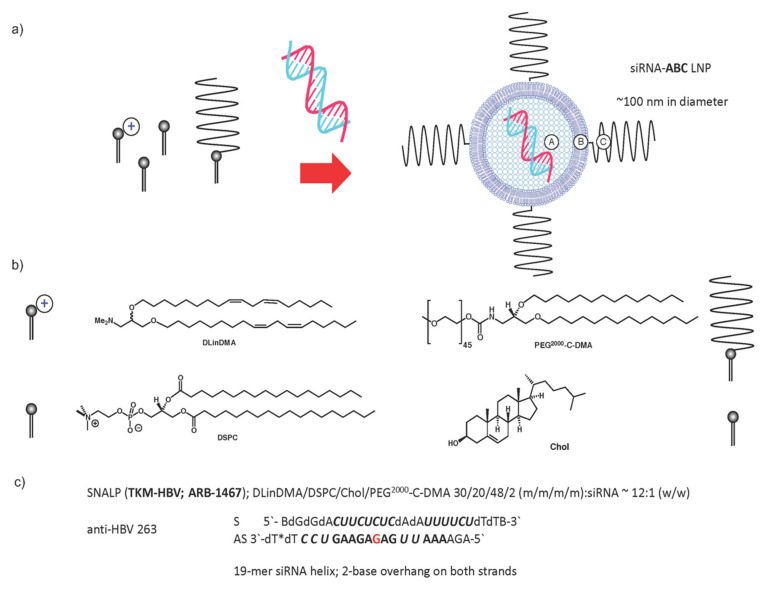
Schematic of TKM-HBV LNPs. The formulation (**a**) of these siRNA-ABC LNPs results from a combination of siRNA-API anti-HBV 263 and lipid self-assembly, involving one ionizable, two neutral, and one PEGylated lipid, as shown in (**b**). In (**c**), the mol fractions of each lipid combination are indicated, as is the approximate ratio of lipid to siRNA in the fully formulated ABC LNP. The structure of anti-HBV 263 is highly chemically-modified. Chemical modifications are indicated as follows: dN are 2′-deoxynucleoside residues (dT: 2′-deoxythymidine; dU: 2′-deoxyuridine; dC: 2′-deoxycytidine; dA: 2′-deoxyadenosine; dG: 2′-deoxyguanidine), B is 3′, 5′inverted deoxy abasic residue, bold italic letters are 2′-F residues, bold letters are 2′-OMe residues. The red letter denotes the putative nucleoside cleavage site residue position (10) in the antisense strand (AS) that acts as a guide strand for guiding the process of RNA silencing while the complementary sense strand (S) acts just as a passenger strand.

**Figure 6:**
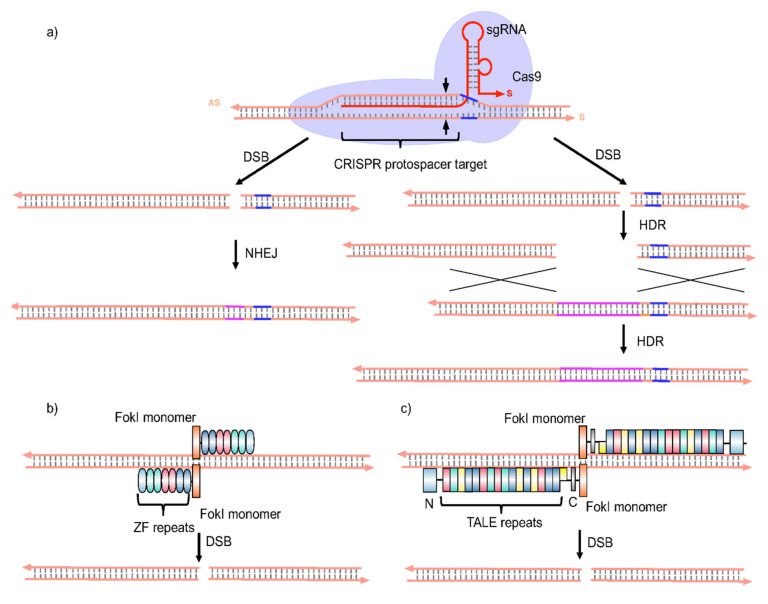
Schematic representations of gene editing technologies. (**a**) Class 2 clustered regularly interspaced short palindromic repeat (CRISPR) systems are part of an adaptive immune system in bacteria Due to comparative simplicity and adaptability, CRISPR has rapidly become a popular genome engineering approach. CRISPR-associated endonuclease (Cas protein, typically Cas9 from *S. pyogenes*) is a nuclease capable of creating targeted double-strand breaks (DSBs) when directed to a given DNA locus by means of a guide RNA (gRNA or sgRNA, red strand in figure). The sgRNA is a short stretch of synthetic RNA composed of a scaffold sequence at its 3′-end for binding to Cas9 and a user-defined sequence (20–22 nts) at its 5′-end, for binding to the AS strand of a selected CRISPR protospacer target in DNA. This CRISPR protospacer target should be immediately adjacent to a protospacer adjacent motif (PAM) (typically 5′-NGG in the DNA S strand, blue strand region in figure). The CRISPR/Cas9 system operates when Cas9 and the sgRNA form a ribonucleoprotein complex. The sgRNA scaffold sequence interacts with surface-exposed positively-charged grooves on Cas9, and the resulting complex then undergoes a conformational change that enables the sgRNA protospacer to zip-bind with the AS strand of the CRISPR protospacer target sequence in a 3′ to 5′ direction, starting with the gRNA seed sequence (first 8–10 nts). Assuming seed sequence/AS strand complementarity, the sgRNA will then continue to anneal to the end, thereby enabling the Cas9 functional endonuclease domains (RuvC and HNH) to undergo a second conformational change that leads to a double-strand break (DSB) (~3–4 nts upstream of the PAM sequence). The resulting, highly selective DSB is then repaired by one of two general repair pathways; (1) The efficient but error-prone non-homologous end joining (NHEJ), (2) The less efficient but high-fidelity homology-directed repair (HDR). The NHEJ repair pathway causes small nucleotide insertions or deletions (indels) at the DSB site. In most cases, indels result in frameshift mutations leading to premature stop codons within the targeted gene and may result in amino acid deletions, insertions and/or protein loss-of-function at the level of translation. Accordingly, the NHEJ repair pathway can be used to disrupt the open reading frame of a gene and generate a knock-out (KO) allele to a target gene that bears the selected protospacer target sequence. On the other hand, the HDR pathway can be used to integrate a donor DNA sequence into the DSB site to create a precise deletion, substitution, or insertion, that leads either to the correction of a pathologic gene or else the targeted knock-in (KI) of a DNA fragment or new gene of interest. Cys_2_His_2_ zinc fingers (ZFs) are DNA-binding domains that each recognize approx. three bps of DNA. (**b**) Alteration of a small number of amino acid residues in or near an α-helix within this domain can lead to changes in DNA-binding specificity. Engineered zinc fingers can be joined together into more extended arrays capable of recognizing longer DNA sequences. A large number of zinc finger arrays engineered can be fused to a non-specific nuclease domain from the Type IIS FokI restriction enzyme to create zinc finger nucleases (ZFNs). The FokI nuclease functions as a dimer, and therefore two zinc finger arrays must be designed for each target site. Most recent ZFN pairs contain complementary obligate heterodimeric FokI domains. A ZFN pair is shown to produce a highly selective DSB, that is followed by NHEJ or HDR as appropriate. Transcription activator-like effector nucleases (TALENs) have rapidly emerged as an alternative to ZFNs. (**c**) TALENs are similar to ZFNs and comprise a non-specific FokI nuclease domain fused to a customizable DNA-binding domain. The fundamental building blocks used to create the DNA-binding domain of TALENs are highly conserved repeats derived from naturally occurring transcription activator-like effectors (TALEs) encoded for by *Xanthomonas* proteobacteria. DNA binding by TALEs is mediated by arrays of highly conserved 33–35 amino acid residue repeats flanked by additional TALE-derived domains at the amino- and carboxy-terminal ends of a given array. Individual repeats bind a single nucleotide residue of DNA as determined by the identities of two hypervariable residues typically found at amino acid residue positions 12 and 13 in each TALE repeat. TALE repeats with hypervariable residues N & N (green) recognize G nucleotide residues, N & I (yellow) recognize A nucleotide residues, H & D (purple) C nucleotide residues, and N & G (red) T nucleotide residues, respectively. As in b), the FokI nuclease functions as a dimer, and therefore two TALENs must be designed for each target site. A TALEN pair may then produce a highly selective DSB, that is followed by NHEJ or HDR as appropriate.

Advanced therapeutics offer genuine opportunities to overcome current limitations of HBV treatment. Several gene therapy approaches might be employed to treat CHB, including the use of (i) RNA interference (RNAi) effectors, (ii) zinc finger nucleases (ZFNs), (iii) transcription activator-like effector nucleases (TALENs), and (iv) clustered regularly interspaced palindromic repeat (CRISPR)/CRISPR-associated endonuclease 9 (CRISPR/Cas9) systems (Figure 4, Figure 5 and Figure 6). All have the potential, directly or at least indirectly, to disrupt cccDNA function. ZFNs, TALENs, and CRISPR/Cas systems may inactivate or even silence this key replication intermediate/viral reservoir of the HBV replication cycle altogether.

RNAi is an important cellular process by which small interfering RNAs (siRNAs) or micro RNAs (miRNAs) induce gene silencing at the post-transcriptional level by targeting mRNA. From a therapeutic point of view, a key realization was that synthetic siRNAs can be delivered to cells and induce similar if not better gene silencing than siRNAs produced intracellularly (in situ) (Figure 4). Accordingly, such RNAi effectors are powerful tools with which to knockdown genes of interest, including those associated with HBV infection [44,45]. Several research groups have focused on inhibiting HBV replication using various expressed RNAi effectors, including pre-miRNA (pre-miR) mimics and short hairpin RNAs (shRNAs) [45].

Adenovirus- and adeno-associated virus (AAV-)derived vectors system have also been described as an efficient way to ensure transient shRNA expression [45], and a number of promising results were obtained with AAV-delivered shRNAs against HBV replication. However, immune responses to adenovirus vectors are a major obstacle to treating CHB patients this way. Moreover, overexpression of shRNAs from RNA polymerase (Pol) III promoters has been shown to cause cellular toxicity [45].

A much-heralded alternative is to use synthetic RNAi effectors delivered to target cells by means of synthetic nucleic acid delivery system technologies such as lipid-based nanoparticles (LNPs). An advantage of this approach is that chemical modifications of RNAi effectors can be introduced to boost the efficacy of the RNAi mechanism intracellularly. Indeed, the pioneering studies of Morrissey et al. [46] made use of an impressive LNP synthetic nucleic acid delivery system and an extensive use of RNAi chemical modification leading to prolonged suppression of HBV replication in vivo. This siRNA-LNP system has since been evaluated in Phase 1 clinical trials (as TKM-HBV; ARB 1467) and is progressing in Phase 2 (Figure 5) [10].

The value of this particular approach is that a similar LNP system has been developed and is now marketed (Onpattro^TM^–patisiran; ALN-TTR02) for the treatment of a polyneuropathy, known as hereditary transthyretin-mediated amyloidosis, by means of the unambiguous siRNA-mediated transthyretin (TTR) target gene knockdown [47,48]. In addition, ARC-520 has been reported that comprises an equimolar mixture of second generation, liver-tropic cholesterol-conjugated siRNAs (siHBV-74 and siHBV-77) plus an excipient that enables endosomal escape of the siRNAs into the cytoplasm where RNAi occurs. ARC-520 has completed CHB Phase 2 clinical trials together with pre-clinical studies in chimpanzees [49], only to be superseded by JNJ3989 (ARO-HBV) which was shown to bring about 1.3–3.8 log reductions in HBsAg blood levels following three administrations after which HBsAg levels were found to rebound only slowly (>6 months) [50]. These data appear very promising, although it remains unclear how low HBsAg levels should be suppressed and for how long before a functional cure can be claimed. In particular, although serum HBsAg levels do correlate with hepatic cccDNA levels, this correlation becomes suboptimal in HBeAg-negative patients, within whom HBsAg apparently originates from integrated HBV DNA expression [49]. Furthermore, it remains unclear how and in what way anti-HBV infection immunity might also be re-established at the same time to sustain any functional cure. These same questions will need to be answered by an alternative small molecule-conjugated anti-HBV siRNA, namely triantennary-*N*-acetyl galactose (GalNAc) ligand-conjugated siRNA (VIR2218) that was derived from parent ALN-HBV and very recently reported to be entering Phase 1/2 trials in co-administration with NUCs.

Otherwise, the biological properties of synthetic siRNAs may be influenced by chemical modification (see Figure 5). For example, 2′-OMe ribose modifications of siRNA ribose moieties have been reported to reduce off-target effects of siRNA silencing [51]. In addition, the inclusion of both 2′-OMe and 2′-F ribose modifications can strongly promote siRNA silencing efficacy [52]. Other promising modifications include the replacement of ribose by the six-carbon sugar altritol that leads to improved siRNA stability and improved anti-HBV data in preclinical testing [53]. Ultimately, though, the in vivo use of synthetic RNAi effectors against HBV infection is limited by the efficiency with which these RNAi effectors are delivered to target hepatocyte cells by synthetic nucleic acid delivery systems. Therefore, in spite of recent promising anti-HBV data sets in preclinical studies [53], there is now a paramount need for new and much improved synthetic nucleic acid delivery system technologies [54,55,56,57] likely assisted by target-cell receptor-specific ligand-mediated delivery [47,48,55], to improve on the efficiency of siRNA delivery (>10^4^ fold). Should this be achieved, then safe, durable, and cost-effective siRNA treatment of CHB becomes all the more likely. Furthermore, if siRNA design rules can also be developed to supplement those rules already in existence, then the intracellular efficacy and duration of action of siRNA effectors may also be enhanced with benefits for a sustained functional cure. Clearly, such possibilities also depend on whether all the suggested improvements lead to the efficient elimination and/or permanent silencing of cccDNA as well as just mRNA transcripts [58]. One way to achieve this would be by using siRNAs that target HBx mRNA, although the full utility of these RNAi effectors is yet to be fully explored [54]. In addition, it has become particularly clear from very recent anti-HBV infection clinical trials using small molecule siRNA-conjugate delivery systems that the silencing of integrated HBV DNA is needed to optimize CHB treatments [49].

Whilst most RNAi effectors used against HBV in preclinical experiments and clinical trials have been majoritively siRNAs, miRNAs are potentially very promising alternatives (as alluded to above). Many reports have suggested that miRNAs play an important role by modulating HBV replication and host responses. For instance, Zhang et al. [59] have reported that miR-199a-3p and miR-210 inhibit HBV by suppressing HBsAg expression and expression of the pre-S1 region of the HBV genome. Other studies have indicated that miR-122 reduces HBV core-associated DNA levels by targeting the highly conserved pgRNA sequence of HBV [60]. Several cancer-related miRNAs such as miR-15a and miR-16-1 [61], miR-17-92 cluster [62], miR-204, and miR-1236 [63] have been described as potential candidates for anti-HBV miRNAs because of their pivotal roles in inhibiting HBV replication through targeting HBV specific RNAs. Other studies indicate that miR-130a can inhibit HBV replication by targeting the transcription of metabolic regulators such as peroxisome proliferator-activated receptor gamma (PPARγ) [64]. Yang et al. [65] in particular reported that an HBV-encoded miRNA (HBV-miR-3) can control the viral self-replication process itself by directly targeting its own transcript, either by reducing HBc protein expression, modulating the levels of pgRNA, or decreasing the amount of cccDNA replication intermediate (Figure 2). Recently, antiviral activities of miR-302c-3p were studied against HBV replication in vitro and in vivo showing that miR-302c-3p significantly decreases viral cccDNA copy numbers and mediates suppression of both HBV replication and HBsAg production by altering the expression of host factors and attenuating HBV transcription. Indeed, such data suggest that miR-302c-3p represents a potentially very potent API lead with which to treat chronic HBV [66]. On the other hand, studies with miR-802 indicated that overexpression of this miRNA actually promotes overall HBsAg and HBeAg expression, while the inhibition of miR-802 acts to decrease HBsAg and HBeAg expression, so suggesting that miR-802 has a crucial impact on HBV expression and replication, thereby implying that miR-802 might be a novel CHB infection target [67]. Indeed, various other experimental studies have confirmed the involvement of miRNAs in the regulation of HBV replication by targeting the HBV genome and/or also by modulating HBV-related transcriptional factors (TFs) that control HBV promoter or enhancer activities [65]. Accordingly, understanding the molecular mechanisms of miRNAs involved in the regulation of HBV suppression could provide important new insights into the development of new and improved RNAi effectors against CHB infections. Nevertheless, the crucial caveat to the use of all miRNAs in CHB treatment is the same as with siRNAs, the absolute need for effective, functional delivery of the selected miRNA API to target hepatocytes, most likely mediated by means of next-generation synthetic nucleic acid delivery system technologies [54,55,56,57].

The ongoing use of RNAi effectors is impressive and promising, but methodologies with very specific anti-cccDNA effects are likely to be essential to control CHB infections completely. ZFNs are a class of gene targeting reagents widely used to modify the genomes in cells such as in plants [68] fish [69], mice [70], ducks [71] and humans [72]. ZFNs used to target cccDNA ex vivo, have been found to be effective at controlling intracellular cccDNA levels [70]. Subsequent reports suggested that control of cccDNA levels in hepatoma cells could reduce pgRNA levels by 29% using ZFNs able to cleave the HBV core gene [73] (Figure 6).

Furthermore, HBV-specific ZFNs delivered using self-complementary adeno-associated virus (scAAV) vectors were found able to target viral DNA polymerase, core, and X genes resulting in efficient inhibition of HBV replication and suppression of the cellular template for HBV persistence [65]. Alternatively, the use of TALENs has been mooted as a powerful way to inactivate cccDNA. Indeed, Bloom et al. [74] designed TALENs to target the HBs or HBc expressing regions of the HBV genome resulting in reductions in HBsAg production compared with controls in Huh7 cells (Figure 6). Likewise, suppression of HBeAg and HBsAg production by TALENs have been observed with several HBV genotypes [75]. Interestingly, IFNα was able to restore the HBV-suppressed IFN-stimulated response element following the administration of TALENs to target conserved regions of HBV viral genomic DNA [75]. Accordingly, HBV-targeting TALENs appear to be a promising method for future gene therapy/cell engineering applications against HBV.

Most recently, the CRISPR/Cas9 gene-editing system has been proposed as a particularly potent way to mediate the complete removal of cccDNA from infected hepatocyte cells [74,75,76] (Figure 6). More so than ZFNs, and TALENs, the CRISPR/Cas9 system can be reprogrammed by means of guide RNAs (gRNAs) to target DNA sequences for suppression of cccDNA in infected cells [77]. Indeed, certain gRNAs targeting conserved regions of HBV appear to promote substantial suppression of cccDNA and HBV proteins in vitro [77]. In addition, gRNAs targeting S- or X-ORFs region clearly inhibit HBsAg expression in vitro too [77]. The utility of single-stranded adeno-associated viral vectors (ssAAVs) for the delivery of engineered CRISPR/Cas9 of *Staphylococcus aureus* (*S. aureus*), showed an efficient inhibition of HBV replication and mutation of cccDNA in cultured cells [78]. In addition, several studies relate that anti-HBV CRISPR/Cas9 systems efficiently modulate the biodistribution of cccDNA both in vitro and ex vivo [77,79]. Several modifications have been made to reduce the off-target effects with CRISPR/Cas9. For instance, the HBV genome can be cleaved and HBV replication suppressed using an engineered nickase-Cas9 system and a pair of sgRNAs to target the HBV genome [80,81]. This same nickase-Cas9 and multiple pairs of sgRNAs has also been shown to induce large deletions in the HBV genome in vitro [82]. Still, the promise of such data must be tempered by the realization that in vivo data is thin on the ground and off-target effects plus undesirable cytotoxicities are likely to be encountered in vivo. For example, recent data indicate that humans have pre-existing immunities to *Streptococcus pyogenes* and *S. aureus* Cas9 proteins [83]. These facts underline the key issue that, in common with siRNA and miRNA RNAi effectors, there is an absolute need for the successful, functional delivery of ZFNs, TALENs, and/or CRISPR/Cas9 components to liver cells in vivo in order to effect advanced therapy. In each case, this is a major delivery problem waiting to be solved, arguably by means of next-generation synthetic nucleic acid delivery system technologies, once more, such as the LNP systems described above which are in fact capable of delivering in principle therapeutic nucleic acids in vivo that vary in size from siRNAs to plasmid DNAs to artificial chromosomes [54,55,56,57].

### 3.2. Therapies Targeting Innate and Adaptive Immune Responses

Powerful and selective gene therapy CHB treatments are arguably best complimented by a second category of advanced therapeutic approach to HBV infections, namely the modulation of viral-host mediating responses by promoting innate immune responses in and involving infected hepatocytes, and/or reversing T-cell exhaustion. Recently, this concept has been very nicely overviewed [22].

#### 3.2.1. Innate Immunity

The innate immune response plays an important role against restricting the general spread of viral infections and activates efficient adaptive immune responses by pattern-recognition receptors (PRRs) on dendritic cells (DCs), natural killer (NK) cells, and natural killer T (NKT) cells. All these components of the innate immune system are modulated during HBV infections [84]. Toll-like receptors (TLRs), retinoic acid-inducible gene I (RIG-I)-like receptors (RLRs), and NOD-like receptors (NLRs) belong to PRRs that are crucial for sensing viral occurrence plus initiating innate immune responses to limit viral spread [84]. Of the different TLRs, TLR9 detects viral DNA, TLR7, and TLR8 (human) identify single-stranded RNA (ssRNA), and TLR3 recognizes viral double-stranded RNA (dsRNA). Thereafter, important signaling networks involving adaptor proteins, protein kinases (ERK, JNK, p38), mitogen-activated protein kinases (MAPK), and PI-3 kinase (PI-3K), plus various TFs (such as interferon regulatory factors 3, 5, or 7 (IRF3, 5, or 7)), nuclear factor-kappa B (NF-κB), and activator protein 1 (AP-1)], play major roles in mediating responses to the binding of pathogen-related ligands to TLRs. The activation of TFs typically leads to the induction of type I IFNs, pro-inflammatory cytokines, and/or co-stimulators that, in turn, develop anti-HBV effects [85]. In particular, the activation of NF-κB, IRF3, and MAPK in hepatocytes appears to suppress intracellular HBsAg levels strongly, while the activation of innate immune cells by TLRs also stimulates the generation of HBV specific immune responses that can lead to substantial suppression of HBV replication [85]. Other studies indicate that the activation of TLRs promotes the production of IFNα and interferon-beta (IFNβ) that could, in turn, assist the total elimination of HBV infection [85,86]. Otherwise, a particular PRR not mentioned above is melanoma differentiation-associated protein 5 (MDA5), which belongs to the RLR family. MDA5 plays a specific role in viral mRNA recognition, and mRNA levels of MDA5 and RIG-I have been found reduced in CHB patients when compared with healthy controls [86]. Several studies indicate that activation of TFs such as NF-κB and IRF3 can lead to inhibition of type I IFN production, thereby interrupting downstream and upstream effects of HBx mRNA recognition by MDA5 and RIG-I [86]. We note here once more that HBx is a key intracellular HBV protein that acts to interfere with transcription, signal transduction, cell cycle progress, apoptosis, and chromosomal stability in hepatocyte host cells (see Section 1.1).

DCs are nowadays considered to have important roles in inducing immune responses against HBV. Indeed, ADV drug-treated CHB patients exhibit higher numbers of DCs in peripheral blood than are found in controls [87]. On the other hand, when programmed cell death protein 1 ligand (PD-L1) levels are high in CHB patients, DCs help to suppress T-cell activation [87]. Accordingly, blocking the interaction of programmed cell death protein 1 (PD-1) with its ligands (PD-L1 and PD-L2) appears a promising way to boost the antiviral functions of exhausted T-cells [88] (see also Section 3.2.2). By contrast, activation of TLR7 increases plasmacytoid DC (pDC) activities hence promoting adaptive and innate immune responses against HBV [43] (see also Section 3.3). Furthermore, the activation of TLR9 in myeloid DCs (mDCs) and pDCs leads to the induction of NK-cell cytolytic activities in HBV infected patients [88]. Reports also suggest that vaccination of Hepato-HuPBL mice with HBc/HBs peptide carrying pDCs influences the ability of HBV-specific T-cells to neutralize infected hepatocyte cells [88]. Unfortunately, the maturation of DCs does not seem to correlate cleanly with enhanced T-cell activities against HBV infection [88], as also indicated by the fact that the DC-related expression of interleukin 2 (IL-2) production may be decreased during CHB infections [89]. Hence, the modulatory role(s) of DCs in managing HBV infections needs further investigation if these cells are to represent a primary source of anti-HBV therapy.

Both NK and NKT-cells could play significant roles in helping CHB patients to mount major innate immune responses against HBV infection by initiating production of immunoregulatory cytokines interferon-gamma (IFNγ), tumour necrosis factor-alpha (TNFα), transforming growth factor-beta (TGFβ), and interleukin 10 (IL-10)] [84]. The activation and suppression of NK and NKT-cells in response to their respective ligands may be altered in CHB patients [89]. Indeed, reduced NK-cell numbers correlate with suppressed cytolysis and the development of peak viremia in acute HBV infected patients [89]. Natural killer group 2D (NKG2D) receptors are positive (activating) receptors expressed on NK and NKT-cells and CD8+ cytotoxic T-cells. These interact with diverse ligands to activate cytolysis, and control of HBV infections, plus the downstream consequences of HBV infections [90]. Natural killer group 2A (NKG2A) receptors are negative (suppressing) receptors that act to down-regulate activating receptors NKG2D, as well as CD16, and natural cytotoxicity receptors NKp30, and NKp46. Notably, a consequence of PEG-IFNα and ADV combination treatment appears to be the lowering of expression of NKG2A receptors on certain NK-cells (CD56 (dim) NK-cells) thereby promoting NKG2D receptor-mediated effects [90]. Such reduced expression of NKG2A receptors and improved IFNγ production correlates with an enhanced activation of these NK-cells resulting in reduced viral resistance during anti-HBV treatment. 

NKT-cells are known as a subgroup of T-cells expressing the NK-cell surface marker-CD56 and T-cell receptor CD3. These are activated by a variety of lipidic ligands, thereby resulting in control of HBV invasion and the development of enhanced anti-HBV immune responses [89]. Unfortunately, although NKT-cell numbers and cytotoxic functions are maintained in the blood of CHB patients compared to healthy controls, their activation involving IFNγ and TNFα production can be attenuated [89]. On the other hand, the ratio of peripheral blood NKT-cells and T-lymphocytes is gradually increased when CHB patients are treated with PEG-IFNα [22]. Importantly too, NKT-cells have been shown to suppress HBV replication following the injection of NKT-activating ligands systemically to transgenic mouse [89]. In summary, with the reasonable exception of NKT-cells, modulating innate immune responses does not appear currently to be a particularly fruitful way of improving CHB treatment without a great deal more knowledge.

#### 3.2.2. Adaptive Immunity

Another route to harnessing immune responses for therapy has been to provoke or reactivate T-cells to target HBV infected hepatocytes. HBcAg plays an important role in stimulating CD8+ T-cell responses, leading to an induction in cytotoxic effects acting on infected hepatocytes. HBcAg also plays an important role in inducing the production of T-helper 1 (Thl) cytokines, including IL-2 and IFNγ, that promote differentiation of CD4+ T-cells into Th1 lymphocytes [91]. In addition, the production of anti-viral cytokines such as IFNα leads to induction of HBV-specific CD8+ T-cells to promote HBV viral clearance, and low-level secretion of IL-2 from HBV-specific CD4+ T-cells regulates the exhaustion of cytotoxic CD8+ T-cell responses in CHB patients [91]. On the other hand, T-cell associated immuno-inhibitors such as PD-1, cytotoxic T-lymphocyte-associated protein 4 (CTLA-4), T-cell immunoglobulin and mucin domain 3 (Tim-3), plus signaling lymphocyte activation molecule/lymphocyte-activation gene 3 (SLAM/LAC3), have all been shown to be key players in the immunosuppression of anti-HBV responses [91]. For instance, overexpression of PD-1 and CTLA-4 leads to CD4+ or CD8+ T-cell exhaustion or dysfunction during CHB infection [92,93]. However, constant expression of PD-1 on CD4+ T-cells can also lead to lower expression levels of negative receptors, including CTLA-4, Tim-3, and killer cell lectin-like receptor subfamily G member 1 (KLRG1) [93]. Importantly though, blocking PD-1/PD-L1 interactions increases the anti-viral efficacy of HBV-specific T-cells in CHB patients, and HBV-specific CD8+ T-cells will also secrete high levels of supporting cytokines [92]. Therefore, the blocking of PD-1/PD-L1 interactions could be a key strategy for re-energizing HBV-specific T-cells in CHB patients.

Turning to CTLA-4, over-expression has a key role in T-cell exhaustion during chronic viral infections, but blockade of CTLA-4 leads to an increase in the IFNγ-stimulated production of HBV-specific CD8+ T-cells in the periphery and liver tissues [93]. Similarly, suppression of CTLA-4 mRNA in lymphocytes by RNAi induces the upregulation of IFNγ and IL-2 gene expression [94]. Otherwise, CTLA-4 plays a pivotal role in increasing T-cell motility and overrides the T-cell receptor (TCR)–induced stop signal required for stable conjugate between T-cells and antigen-presenting cells (APCs) [95]. Therefore, the blocking of CTLA-4 should restore impaired T-cells and could be another strategy to enable the complete elimination of HBV infections. Otherwise, Tim-3 plays a pivotal role in the death of Th-1 cells and promotes peripheral tolerance [96]. Accordingly, the upregulation of Tim-3 is associated with T-cell exhaustion in CHB infection [96], and significant expression of Tim-3 causes sudden changes in Th-1 responses in CHB patients promoting the persistence of HBV infections [97]. In addition, the high expression of Tim-3 in HBV-specific CD8+ T-cells ensures that these cells are unable to secrete IFNγ and TNFα, and so are unable to undergo any HBV-specific CD8+ T-cell clonal expansion at all [96]. On the other hand, a blockade of Tim-3/galectin-9 interactions will cause an upregulation IFNγ production in peripheral blood mononuclear cells (PBMCs) or NK-cells and enhance cytotoxicity in CHB patients, completely consistent with a role for Tim-3 activation in suppressing anti-viral responses. Indeed, Tim-3 polymorphisms are associated with viral persistency and HCC traits in CHB patients [97]. Accordingly, a blockade of Tim-3/galectin-9 interactions could be an exceedingly useful approach for treating CHB patients.

A completely different approach to harnessing adaptive immune responses for the treatment of CHB involves a combination of ex vivo gene and cell therapy approaches. This approach, known as adoptive cell transfer (ACT), relies on the isolation, for example, of autologous T-cells that are cultivated then subject to virus-mediated genetic modification ex vivo leading to engineered T-cells with refined immune functions. Currently, there are two ACT products on the market created by isolation, cultivation, and engineering of patient T-cells to express chimeric antigen receptors (CARs), after which the resulting transgenic “CAR-T” cell lines are returned to the same patient for disease treatment by immunotherapy. The first product is known as KYMRIAH™ that targets leukaemia cells for destruction in cases of B-cell precursor acute lymphoblastic leukaemia (ALL), diffuse large B-cell lymphoma (DLBCL), and/or high-grade B-cell lymphoma and DLBCL arising from follicular lymphoma. The second product is YESCARTA™ for the treatment of primary mediastinal large B-cell lymphoma, high-grade B-cell lymphoma, and/or DLBCL.

Recently, ACT was shown to have promise in treating HBV in vivo in a mouse model of HBV infection [98]. CD8+ T-cells were isolated then genetically engineered by retrovirus transfection to express specific CARs that bind HBV envelope proteins (S-CARs), resulting in S-CAR-T cell lines. The major difference between TCRs and CARs is that TCRs are typical human leukocyte antigen (HLA)-restricted heterodimer receptors that bind MHC-peptide complexes found on the surfaces of T-cells, while CARs are single-chain antibodies that bind HBV antigens independently of HLA restriction. After reintroduction, the S-CAR-Ts were found to locate efficiently to hepatic tissues and efficiently control HBV replication compared with controls, and only caused transient liver damage in mice in vivo. A large amount of circulating viral antigen did not appear to appear or over-activate the S-CAR-Ts [98]. More recently, when an HCC patient with chemoresistant metastases expressing HBV antigens was treated with autologous T-cells engineered to express an HBsAg-specific TCR, then significant reductions in HBsAg levels were observed without exacerbation of liver inflammation or other obvious toxicity [99], although clinical efficacy was not established in this case. Thus CAR-Ts and TCR-engineered T-cells are promising as agents to counter HBV and associated diseases; extensive trials in CHB patients are now essential. Unfortunately, the costs of such immunotherapies are currently quite prohibitive for widespread use.

### 3.3. Therapeutic Vaccinations

Currently, therapeutic vaccinations are focused on restoring diminished T-cells responses against HBV antigens [100,101]. However, the major problem with therapeutic vaccination is the inability to break tolerance. Adenovirus-based vaccines have often proven safe and immunogenic however, pre-existing adenoviral immunity limits their use. For example, the recombinant adenovirus (rAd) vaccine TG-1050 (T101) targeting HBV proteins has been shown to induce anti-HBV immunogenicity resulting in good safety, tolerability, producing both IFNγ and TNFα while stimulating cytolytic functions in HBV persistent mice established by infection with a recombinant AAV vector carrying the HBV genome [100]. Following this, early phase clinical trials with TG1050 demonstrated HBV-specific T-cell responses sufficient to trigger a mean 0.45 log decrease in HBsAg levels at day 197, although anti-rAd antibodies were also observed [10]. As an alternative, a recombinant HBV (rHBV) vector has been reported comprising a modified viral core gene that specifically delivers a foreign antigenic polyepitope to the liver following hydrodynamic injection. The expression of the foreign antigenic polyepitope in the hepatocytes of HLA-A2/DR1 transgenic mice appeared to attract/reactivate a vigorous intrahepatic T cell response against HBsAg without causing major liver injury. Following this, a mouse model of HBV persistence was first established by infection with a recombinant adeno-associated virus (rAAV) carrying the HBV genome (rAAV8-HBV), thereafter vaccination was performed with rAd loaded with rHBV (rAd/rHBV) and found to elicit a foreign-antigen-specific T-cell response sufficient to trigger effective viral clearance with seroconversion [102].

DNA-based vaccinations of CHB patients have held interest for a while [101]. Nucleic acid-based-vaccines of this type are, in principle, able to induce cellular and humoral immune responses in contrast to the situation with protein-based vaccines that only induce humoral/antibody responses [101]. For example, when a DNA vaccine coding for S and preS2 domains of the HBV envelope proteins were administered to CHB patients, the amount of HBV DNA in serum was reduced, levels of HBV-specific IFNγ-secreting T-cells were increased, and anti-HBcAg responses were increased as well. Furthermore, other DNA-based vaccinations have been shown to stimulate an increase in the level of activated CD4+ and CD8+ T-cells, thereby boosting cellular immune responses [101]. Otherwise, one recent DNA-based vaccine candidate is INO-1800 (RG-7944), comprising plasmid DNAs coding for HBs and HBc. INO-1800 alone or in combination with INO-9112 (DNA plasmid expressing interleukin 12 [IL-12]) has successfully entered early-stage clinical trials administered by electroporation to CHB patients also receiving NUC treatment. In all likelihood, current DNA-based vaccination strategies are likely to superseded by mRNA-based vaccination strategies going forward.

Turning to other vaccination approaches, protein or peptide-based vaccines can induce high titers of anti-HBV-specific antibodies, but they can require continuous dosing and only induce weak cellular immune responses [101]. An early such vaccine was GenHevac B Pasteur, comprising recombinant HBs and Pre-S2 antigen, which was shown to provide long-lasting immunity against HBV by reducing levels of circulating HBV virions post-administration in serum, when used along with adjuvants such as aluminum hydroxide. Much more recently, ABX-203, a combination of recombinant HBsAg with HBcAg, administered subcutaneously or in a nasal spray, was reported to exhibit immunogenic effects including HBs-specific T-cell responses in Phase 2 clinical trials as an adjunct to NUC therapies [103]. Otherwise, given the need to address innate immunity to control HBV infections a number of alternative vaccination strategies are being invoked in parallel. For example, GS-9620 was developed and tested in chimpanzees as a small molecule agonist for dendritic cell receptor (DCR) TLR7s so as to activate DCs to act as innate immune cell-like vaccines [104]. Unfortunately, in CHB clinical trials, GS-9620 failed to induce significant changes in HBsAg levels, although responses in HBV-specific T-cells and NK-cells were notably increased in CHB patients also treated with NUCs [10]. On the other hand, GI-13020 (GS-4774) is a novel heat-killed recombinant yeast vaccine expressing an HBx-HBs-HBc chimeric protein, that was found to induce both CD4+ and CD8+ T-cell responses while being reportedly initially well-tolerated in the clinic. However, in more recent Phase 2 clinical trials with CHB patients, GS-4774 administered with the NUC TDF, was not shown to invoke clinically significant reductions in HBsAg although the vaccine did appear able to break immune tolerance in CHB patients, and so might be used in combination with other antiviral agents to boost anti-viral immune responses [105]. Overall, although many of the above-mentioned vaccines have been shown to be potentially attractive means to treat CHB patients, vaccination side effects in the clinic remain problematic. Also, innate immunostimulation is frequently inadequate. Therefore, potent bespoke adjuvants should be included realistically as part of future vaccination protocols, in order that specific immune responses are substantial enough to render therapeutic vaccination sufficiently potent.

## 4. Moving towards Precision Medicine Approaches for CHB Treatment

Broadly, speaking precision medicine can be considered the most desirable future for medicine. This can be broken down into two main fields:

1. Personalized medicine: This means understanding the genetic, immunological and/or metabolic individuality of patients in order to match individual patients with the most appropriate APIs for treatment of disease in these patients, i.e., “one size does not fit all.”
2. Precision therapeutics: This means taking control of the delivery of APIs to target and/or selecting APIs for use with extreme target specificity.

The contributions and prospects for personalized medicine in CHB treatment and for precision therapeutic approaches for the treatment of CHB will be considered in the following.

### 4.1. Pharmacogenomics Studies

Currently, data from the Human Genome Project, genome-wide association studies (GWAS) and other pharmacogenomics studies are being correlated for use in personalized medicine in various fields. Indeed, GWAS studies across complete genomes have now led to the characterization of several potential genetic factors influencing the pathogenesis of HBV-related disease traits [106]. These genetic factors are linked to the substantial variations in anti-HBV immune responses observed in patients infected with HBV. These variations literally extend from asymptomatic self-limited infection, inactive carrier states, chronic hepatitis, cirrhosis, end-stage HCC, all the way to liver failure. Such variations clearly must have an influence upon the effectiveness of therapeutic interventions and, unless understood, represent real potential obstacles to effective treatment [106].

Investigations into genetic factors linked with substantial variations in anti-HBV immune responses began with studies in Japanese populations. Kamatani et al. were the first to report two single nucleotide polymorphisms (SNPs) loci, namely rs3077 within the HLA class II gene HLA-DPA1 and rs9277535 within the HLA-DPB1 gene, both associated with CHB [107]. Subsequently, Mbarek et al. identified two other CHB linked SNP loci, namely rs2856718-A within the HLA-DQB1 gene, and rs7453920-G within the HLA-DQB2 gene [108]. At the same time, in data from Han Chinese populations, Liu et al. identified rs11866328 G, located in the GRIN2A gene within region 16p13.2 as a CHB susceptibility locus associated with disease progression in HBV carriers [109]. Subsequently, Hu et al. reported that the SNP locus rs3130542-A in gene TRNAI25, within region 6p21.33 (near the HLA-C), and rs4821116-G in gene UBE2L3, within the region 22q11.21, are also associated with an increased occurrence of CHB in Han Chinese populations [110]. More recently. Kim et al. found that the SNP locus rs652888 in the EHMT2 gene, and the SNP locus rs1419881 in the TCF19 gene, within HLA regions, are associated with an increased risk of CHB occurrence. Furthermore, Kim et al. also verified that SNP loci rs9277535, rs3077, rs7453920, and rs2856718 (mentioned above) are indeed all associated with the increased occurrence of CHB in both Japanese and Korean populations [111]. Most recently, SNPs associated with CHB progression were confirmed by GWAS in the HLA-DPA1 and HLA-DPB1 genes of these HLA class II genes [112]. In other GWAS studies, Chang et al. separately described three SNPs within HLA-DPB1 (rs9277535), HLA-DQB2 (rs7453920), and HLA-DPA3 (rs9366816) loci as being independently associated with the persistence of HBV infections in male Taiwanese Han Chinese populations. Chang et al. further concluded that HLA-DPB1, HLA-DQA2, and HLA-DQB2 genes are associated with persistent HBV infection in male Han Taiwanese, and suggested how the HLA-DQA2 and HLA-DQB2 complex might actually represent a key protein complex that modulates therapeutic responses to CHB infection [113].

In alternative GWAS studies, the extent to which five separate SNP loci (rs2188971, rs8103163, rs7248488, rs2188972, and rs8105767) in the zinc finger gene (ZNF208) might be associated with HBV risk has been studied. ZNF208 polymorphisms play complex roles in the development of HBV, accordingly, an association was studied between SNPs, haplotypes of ZNF208, and the risk of contracting HBV infections [109]. Data suggested that SNP locus (rs1883832) might be a valuable predictive factor for CHB patients with HBeAg seroconversion. So too, the presence of SNP loci (rs9277535) at HLA-DPB1 might also be a valuable predictive factor for CHB in HBeAg-negative patients, however further verifications of this conclusion are recommended due to study limitations [110]. Another GWAS based study involving Japanese CHB patients, with and without HCC, has led to the identification of SNP loci in the HLA class I region (in HLA-DR, -DQ, and -DP genes) associated with HBV-related HCC disease progression [114].

Overall, GWAS studies to date have led to the identification of a range of SNP loci apparently associated as genetic risk factors for CHB either alone or in response to drugs. Therefore, once the role of each locus has been identified, then a solid theoretical foundation can be assembled for early diagnosis and prevention of CHB, plus treatment. As the first steps in this direction, Pan et al. [115] performed GWAS studies to identify genetic factors that might underlie the variation in the immune response to HBV vaccinations (low, intermediate, and high). In this case, they demonstrated that two SNP loci (rs3135363 and rs9277535) in the HLA-DPA1 and HLA-DPB1 genes had significant effects on antibody responses, suggesting that HLA-DP region gene variants may contribute to both HBV persistence and non-responsiveness to vaccination [115]. Another such GWAS study conducted with Chinese adults led to the identification of another SNP locus (rs477515) within the HLA-DR gene region associated with non-responsiveness to HBV vaccination, suggesting that HLA-DR might be a critical susceptibility gene region modulating hepatitis B vaccine-induced immunity [115]. In a follow up to this, Wu et al. [116] reported that SNP locus rs7770370 was the most significant genetic factor modulating responses to hepatitis B booster vaccination and that nearby SNPs might contribute to the long-term immunological memory against HB vaccination. Thereafter, three SNP loci (rs7770370, rs9277535, and rs3077) within the HLA-DP gene region were found independently associated with non-responsiveness to vaccination in Korean infants and Japanese medical students [117,118]. Similarly, in Chinese populations, the SNP locus rs477515 in the HLA-DRB1 gene was found linked to HBV persistence and vaccination non-responsiveness [119]. Overall, although several SNP loci have been identified and associated with HBV persistence and non-responsiveness to vaccination, studies have yet to be extended to achieve a fuller understanding of biological mechanisms involved, which will be necessary in order to provide a solid foundation with which to develop therapeutic and vaccination strategies sufficient to enable total HBV clearance in CHB patients.

### 4.2. Next-Generation Gene Sequencing

The use of next-generation sequencing (NGS) has become an established method for virus detection. To date, NGS has been instrumental in the discovery of novel viruses and the characterization of viral communities. Initially, NGS was used to discover a new arenavirus [120], a new Ebola virus [121], and Zika virus [122]. NGS is also applied for the characterization of viruses in situ, in animals [123] and in humans [124]. Recently, NGS was used to detect pre-S mutants in the plasma of HBV-related HCC patients, suggesting that NGS may provide better accuracy for the detection of pre-S deletions, potentially improving prediction outcomes for patients with HBV-related HCC [125]. NGS analysis was used to assess HBV mutations and the relevance of mutations to HCC development. In addition, ultradeep sequencing was used to examine the diversity between intrahepatic HBV strains and those circulating in the serum [125]. This same technique was also used for the analysis of HBV reverse transcriptase quasi-species heterogeneities, to identify not only those host genes that are frequent sites for HBV integration but also to study the effects of HBV integration on the genomes of HCC patients [126]. NGS data has the power to complement GWAS and other pharmacogenomics data sets with data on genotyping, to account for drug resistance or variable responses to treatment for vaccine development, efficacy monitoring, and for characterization of the metagenome [127]. All this is possible due to the increased sensitivity of NGS and its ability to provide comprehensive and detailed patient information. Once again, NGS studies have yet to be extended to achieve a fuller understanding of the biological mechanisms that underlie HBV survival post-infection and prolongation of CHB.

### 4.3. Mass Spectrometric Studies

Mass spectrometry (MS)-detection represents an alternative to NGS working at the level of proteomics and genomics. MS is cost-effective and easy to handle and has been applied successfully for bacterial disease typing and cancer marker detection. Currently, Matrix-assisted laser desorption/ionization (MALDI) and surface-enhanced laser desorption/ionization (SELDI) connected with time-of-flight (TOF) detector as well as electrospray ionization (ESI) have been widely used system for the clinical proteomics [128]. In addition, MS analysis systems have also been found applicable for routine surveillance of viral infections.

In the case of HBV infections, high-throughput MALDI-TOF MS has been used successfully to study 60 drug-resistant HBV variants, leading to the identifications of mutations with high levels of accuracy and low detection limits [129]. In addition, magnetic beads (MBs) have been used with MALDI-TOF MS to investigate protein profiles, and serum biomarkers of HBV infected patients with or without HCC. This strategy enabled the definition of optimal protein profiles for discrimination between HBV infected patients with or without HCC, and so identified effective serum biomarkers and diagnostic models for HCC infection at the molecular level [130]. In parallel, a MassARRAY system for nucleic acid analyses by MALDI-TOF MS was adapted for HBV genotyping as well. This MassARRAY system generates MS patterns that are automatically compared with MS patterns simulated according to sequences derived from known HBV genotypes. This approach is capable of detecting wild type and mutant alleles and can identify minority genotype combinations even if a given minority is present <10% [131]. The sensitivity of MS methods for detecting HBV genotypes is impressive.

Earlier, MALDI-TOF MS-based methods were used to detect as few as 100 HBV copies of HBV genome in the liver cells of 40 CHB patients, and sequencing data were analyzed to determent the ratios between HBV wild type to variant oligonucleotides encoding mutations to the YMDD domain in viral DNA polymerase. Data indicate the importance of mutations in the YMDD domain, given that LMV treatment of patients with mutations in the YMDD domain can trigger acute liver failure [132]. Given that variations in the HBV genome can cause significant problems for the selection and use of antiviral therapies that may lead post-treatment to drug resistance or affect virus replication, then such data is invaluable in the context of personalized medicine approaches to CHB treatment. Interestingly, MS studies are already giving a fuller understanding of biological mechanisms involved with HBV infection, with implications for improved CHB treatment. MS-based MALDI-TOF MS approaches are able to account for drug resistance to antiviral therapy and diagnose resistance to treatment based upon HBV genotype variations present, even at different levels of relative abundance.

### 4.4. Diagnosis of HBV Infections

Traditional diagnostic techniques remain the mainstay for the diagnosis of HBV infection. However, there are moves to use rapid diagnostic tests (RDTs) that aim to accelerate the use of laboratory-based immunoassays such as traditional radioimmunoassays (RIA), enzyme immunoassays (EIA), an HBsAg serological test, and a nucleic acid amplification test (NAT) for the serodetection of HBV DNA viral loads [133]. RDTs have been shown to be sensitive for the detection of HBsAg mutants, where ELISA assays failed. Recently, other new diagnosis tools have been created for the screening, diagnosis, and optimal patient management of HBV infections using electrochemiluminescence immunoassays (ECLIA), microparticle enzyme immunoassays (MEIA) and chemiluminescent microparticle immunoassays (CMIA). All these detect HBsAg levels in the blood by using signal amplification [134]. Otherwise, there are quantitative DNA detection methods such as ultraviolet (UV) spectrophotometry, real-time polymerase chain reaction (PCR), digital PCR, isothermal amplification methods, and biosensors for the detection of HBV DNA, its antigens or anti-HBV human antibodies [135]. Good examples include electrochemical biosensors able to detect HBV DNA down to nM concentration levels and a piezoelectric biosensor for HBV DNA based on the mass-transducing function of a quartz crystal microbalance (QCM) [136]. Very recently, a new method of detecting cccDNA by cccDNA-selective droplet digital PCR (ddPCR) methods was used to detect HBV cccDNA in serum, single cells, and preserved tissue samples [137]. Circulating RNAs (see Section 1.1) have the interesting potential clinical utility for quantification of HBV RNA in serum and are also being developed as alternative biomarkers with which to monitor viral persistence and the progression of liver disease.

Another aspect of HBV diagnosis is the use of microarray technologies. Microarray technologies have characteristics and performance levels that are highly useful for clinical, epidemiological, and research-level studies of HBV infections. Multiple microarray platforms exist, including printed double-stranded DNA and oligonucleotide arrays, in situ-synthesized arrays, high-density bead arrays, electronic microarrays, and suspension bead arrays. For example, Gauthier et al. [138] reported on the use of a DNA microarray technology to diagnose and correlate HBV genotypes and mutations (S, Pol, Core, and X genes). They performed microarray analysis combined with PCR to amplify whole virus genomes leading to the identification of 994 mutations at 298 positions making this array a comprehensive tool with which to monitor disease evolution and treatment efficacy [138]. More recently, microarray analyses were performed in 620 Chinese patients with CHB infection, revealing that NUC resistance in HBV-positive patients hospitalized in China was low enough to obviate the need for NUC resistance testing in advance of treatment. In addition, it concluded that CHB patients infected with genotype C possessed greater numbers of NUC-resistant mutations than CHB patients infected with genotype B virus [139]. Developments such as these in microarray technologies have been matched by important developments in microfluidic technologies. Microfluidics are often known as “lab-on-chip” technologies capable of sample and reagent processing as rapid micro total analysis systems. Microfluidics devices have been used to study HBV detection, replication, and genotyping [140].

At this stage, none of the current diagnostic technologies used for HBV infection can be described as advanced diagnostics that make use of imaging modalities such as MRI, computed tomography (CT), or nuclear medicine imaging such as positron emission tomography (PET) or single-photon emission computed tomography (SPECT). Arguably, the use of such techniques might not only enhance our understanding of CHB disease aetiology and pathology, but would be a very useful foundation for image-guided approaches to CHB treatment, in particular putative precision therapeutic approaches (PTAs) for CHB treatment that are briefly referred to below. Therefore, we anticipate a rising need and interest to use advanced diagnostics for the detection of CHB and for monitoring disease progression as a function of time and treatment modalities.

## 5. Where to Next and Why? The Future of HBV Treatment

**Figure 7:**
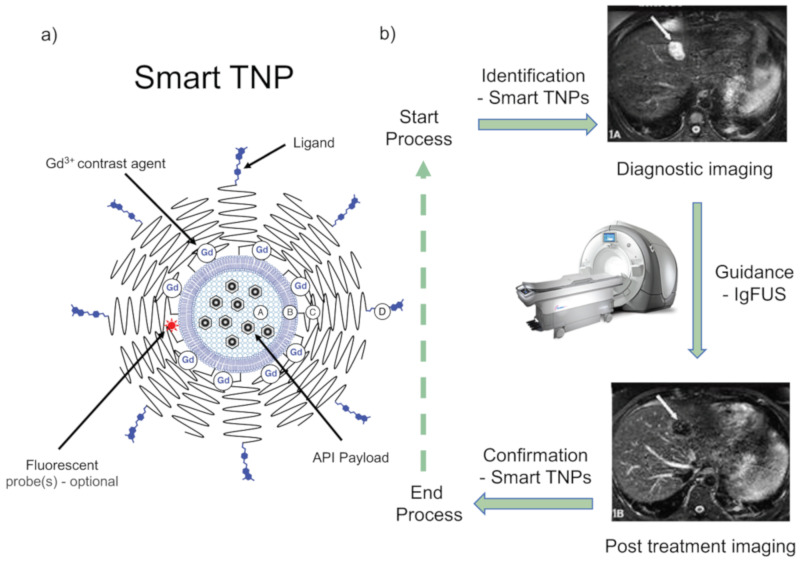
Schematic representation of a possible PTA for the treatment of CHB in patients. (**a**) A schematic representation of a theranostic lipid-based nanoparticle (smart TNP) that is enabled for the targeted delivery of an appropriate therapeutic API. According to this paradigm, a smart TNP, as shown, comprises a therapeutic API payload for delivery (A) that is encapsulated within concentric layers of lipids (B) and a layer of stealth-biocompatibility polymer (C) (typically polyethylene glycol), then capped with optional biological cell receptor-specific targeting ligands (D) on its surface. For real-time/diagnostic imaging purposes, a given API should be co-delivered with either a near-infrared fluorophore and/or an MRI contrast agent such as chelated-Gd^3+^ ions—which act as a positive contrast agent in images of body sections generated by MRI. (**b**) Any PTA for CHB treatment implies a three-stage process; (1) identification of diseased areas of the liver using a clinically appropriate advanced imaging technique, such as MRI, in conjunction with targeted imaging agents (including imaging lipid nanoparticles [LNPs], or smart TNPs, as above) to show precisely where priority HBV infections of hepatocytes are found; (2) guidance of smart TNPs to high priority HBV infection areas by means of image-guided focused ultrasound (IgFUS) typically aided by MRI; (3) confirmation of effects of targeted API delivery on the zones of CHB infection in the liver by long-term follow up using a clinically appropriate advanced imaging technique, such as MRI. In this instance, smart TNPs will label up disease target areas for as long as infected cells or intracellular infection exists. The schematic anticipates that there may be more than one round of treatment as appropriate.

CHB disease management is immensely complex. Without doubt, current anti-HBV treatments are efficient in reducing viral loads but insufficient for functional cure. NUCs and PEG-IFNα are able to suppress viremia but these agents suffer major limitations from virus resistance and the presence of API toxicities if used for life long treatment [141], even if used in combination. Undoubtedly, current treatments do prolong patient survival rates, mainly through suppression of viral replication. However, the failure of these current treatments ensures that progression to either HCC or LC is probable either post-treatment or during treatment as drug resistance takes hold [141]. Clearly, there is a need to do much better. With reference to the HBV replication cycle,(see Section 1.1), ultimate treatments for CHB infection should result in the total silencing of integrated HBV DNA, the eradication of cccDNA, control of the transcription of cccDNA in infected hepatocyte cells, and the induction of a meaningful anti-HBV immune defense, all to ensure full serum clearance of HBV DNA, HBsAg, and hence functional cure of CHB patients. In this case, the route forward should involve two main aspects:

1. A commitment to improve fundamental understanding of the viral replication cycle, viral persistence, viral minichromosomal formation, viremia, HBeAg-positivity, identification of cellular receptors, other host –virus interactions, and viral genome heterogeneity (HBV genotype variation). All this to be made possible by using the very best possible cell and animal model systems. So too, studies on interactions with host immune responses must go hand in hand with basic research into underlying mechanisms, as mentioned above. Such an effort should result in more effective antiviral strategies that target different stages of HBV replication and restore host adaptive immune responses. Knowledge gained may then be used to devise combination therapeutic approaches that target CHB infection. In CHB infection, it is likely that combination therapy will be needed to obtain a functional cure. In recent years, various therapeutic approaches have been devised to suppress cccDNA formation and/or propagation as a primary and strategic way to overcome HBV persistence in vivo [142]. However, this remains a key unrealized goal that needs to be meet as a matter of high priority. Moreover, the growing consensus is that optimal API combinations, that would best achieve functional cure of CHB infections, that would best achieve functional cure of CHB infections, are combinations of API(s) that target the virus replication cycle and agents that target the immune system [10,22].
2. A potential commitment towards PTAs for CHB treatment. As noted above, precision therapeutics means taking control of the delivery of APIs to target and/or selecting APIs for use with extreme target specificity. Accordingly, a PTA for CHB treatment would have the following basic elements (Figure 7) [56,143]:
  - Identification of infected liver cells in situ, potentially achieved through the application of advanced diagnostic imaging techniques such as magnetic resonance imaging (MRI), near-infrared (NIFR) fluorescence, or resonance Raman, in appropriate combination with imaging nanoparticles (or theranostic nanoparticles (drug-delivery combined with imaging)).
  - Guidance of theranostic nanoparticles to infected cells, potentially made possible by the exogenous application of a tissue irradiation technique, such as image-guided focused ultrasound (IgFUS), to direct theranostic nanoparticles (TNPs) to accumulate in infected cells [143], so as to clear infection there. Infected-cell receptor-specific targeting ligands may also be attached to TNPs in order to facilitate this process.
  - Confirmation of therapeutic effects—potentially made possible by the accumulation of TNPs into infected cells. Infected cells should be visible for as long as infection continues.

PTAs are essentially image-guided approaches to treatment with diagnostic real-time imaging realized by the targeted accumulation of imaging nanoparticles (or theranostic nanoparticles) to cellular sites of infection. Thus far, some imaging nanoparticle systems have been reported for the imaging of liver diseases [144]. For example, gold nanoparticles (AuNRs) have been used to develop a biosensor for detecting HBsAg in biological samples or specimens such as buffer, blood serum and plasma [145]. However, the detection of infected hepatocytes in situ has not yet been achieved routinely, so a comprehensive PTA for CHB treatment is currently a future opportunity rather than a present reality, but not for long, we would venture to hope.

Overall, the treatment of CHB remains a major challenge and an area of unmet medical need. Current antiviral drug treatments are adequate but far from ideal given the extent of this unmet medical need. In our opinion, the treatment of CHB represents a huge opportunity for advanced therapeutics, in combination with advanced diagnostic techniques to make possible PTAs for CHB treatment (as outlined above). Such treatment regimes should also operate alongside personalized medicine approaches for treatment, correlating the use of a therapeutic approach and API according to personalized infection and patient medical data.

## Figures and Tables

**Table 1:** Characteristic features of HBV genotypes, sub genotypes and geographical distribution.

Genotypes	Sub-Genotypes	Serological Serotypes	Mode of Transmission	Geographical Distribution
A	A1, A2, A3	adw	Patients most at risk of chronicity are those infected during early life (neonates and children). Adults are infected through sexual contact	Europe, North America, Sub-Saharan Africa and Western Africa
B	B1, B2–B5, B6	adw, ayw	Perinatal (during childbirth associated trauma) —most common or vertical (via the placenta)—less common	Asia
C	C1–C3, C4, C5, C6–C11	adw, ayr, adr	perinatal or vertical	Asia
D	D1–D6	ayw	Patient infected through homosexual or or bisexual or heterosexual contact	Mediterranean area, Middle East and India
E	NA	ayw	Horizontal and homosexual	Sub-Saharan Africa and some other continents
F	F1–F4	dw	Horizontal	Central America
G		dw	Horizontal	France, Germany, United States and Mexico
H		dw	Horizontal	South America
I	I1, I2	dw	Pariental/horizontal	Vietnam and Laos
J		dw	Horizontal	Japan

**Table 2:** Summary HBV genotype responses to CHB treatment by IFNα or PEG-IFNα and NUCs.

Genotype	Response to Treatment		Reference
	IFNα or PEG-IFNα	NUCs	
A	weak responder to IFNα; strong responder to PEG-IFNα	strong responder to NUCs; drug resistance noted	[21,22]
B	strong responder to IFNα; strong responder to PEG-IFNα	strong responder to NUCs; drug resistance noted	[21,22,23]
C	weak responder to IFNα; weak responder to PEG-IFNα	weak responder to NUCs	[21,23]
D	weak responder to IFNα; weak responder to PEG-IFNα	weak responder to NUCs	[21]
E	strong responder to IFNα; weak responder to PEG-IFNα	adequate responder to NUCs	[24,25]
F	weak responder to IFNα	adequate responder to NUCs	[24]
G	stronger response to IFNα	adequate responder to NUCs	[24]
H	weaker response to IFNα	adequate responder to NUCs	[24]
I			[25]
J			[25]
